# Fluorescence-Guided Spatial Mapping of p21-Expressing Senescent Cells in Aged Liver at Single-Cell Resolution

**DOI:** 10.3390/biom16040579

**Published:** 2026-04-14

**Authors:** Jer-En Hsu, Binsheng Wang, Yongha Hwang, Weiqiu Cheng, Qingyang Zhao, Yichen Si, Ming Xu, Hyun-Min Kang, Jun Hee Lee

**Affiliations:** 1 Department of Molecular & Integrative Physiology, University of Michigan, Ann Arbor, MI 48109, USA; jerenhsu@umich.edu (J.-E.H.); yonghah@umich.edu (Y.H.); qingyanz@umich.edu (Q.Z.); 2 Masonic Institute on the Biology of Aging and Metabolism, University of Minnesota, Minneapolis, MN 55455, USA; wangb@umn.edu (B.W.); mixu@umn.edu (M.X.); 3 Department of Biochemistry, Molecular Biology and Biophysics, University of Minnesota, Minneapolis, MN 55455, USA; 4 Space Planning and Analysis, University of Michigan, Ann Arbor, MI 48109, USA; 5 Department of Biostatistics, University of Michigan, Ann Arbor, MI 48109, USA; weiqiuc@umich.edu (W.C.); ycsi@umich.edu (Y.S.)

**Keywords:** aging, spatial transcriptomics, Seq-Scope, cell senescence, p21, Gpnmb

## Abstract

Aging is a regulated process marked by the accumulation of senescent cells, which remain viable but no longer divide. Senescent cells contribute to age-associated phenotypes and diseases, including osteoarthritis, dementia, and cancer, but their scarcity and heterogeneity have limited study. Here, we developed a fluorescence-guided high-resolution spatial transcriptomic profiling approach to precisely locate and profile p21-reporter-positive cells in aged liver. This method enabled unbiased detection of a p21-associated, senescence-enriched cell population and revealed its diverse cellular identities, including hepatocytes, macrophages, neutrophils, and plasma cells. Our analysis further showed that activated macrophages and hepatic stellate cells were more likely to exhibit a p21 positive (p21^+^) state than their resting counterparts. Transcriptomic profiling of p21-expressing cells indicated heterogeneous senescence-associated secretory phenotype (SASP) programs, with distinct inflammatory and remodeling signatures across cell identities and their spatial positions. In parallel, we identified an aggregation of interferon-stimulated gene (ISG)-expressing cells with limited overlap with p21 positivity, suggesting a distinct aging-associated stress program. Taken together, our fluorescence-guided spatial transcriptomic framework enables high-resolution, single-cell mapping of senescence in situ, delineating both senescent cell type specificity and cell identity–independent senescence programs, thereby advancing a more comprehensive understanding of regulatory mechanisms underlying aging.

## 1. Introduction

Cellular senescence is a regulated aging process in which cells remain viable yet undergo stable cell-cycle arrest and acquire metabolic changes that can reshape tissue homeostasis [1,2]. With age, senescent cells accumulate and have been implicated in diverse age-associated pathologies, in part through paracrine signaling mediated by senescence-associated secretory phenotypes (SASPs) [3,4]. A persistent challenge is that senescent cells are typically rare, heterogeneous, and context dependent, causing difficulties in identifying them and defining their tissue-level interactions [5].

Among senescence-associated programs, upregulation of the cyclin-dependent kinase inhibitor p21 (*Cdkn1a*) is widely used as a hallmark of senescence and a key effector of cell-cycle arrest, acting downstream of damage and stress signaling to enforce growth inhibition [6]. The accumulation of p21 positive (p21^+^) cells has been identified in aged tissues in vivo, and clearance of this population in rodents has been shown to both extend lifespan and improve healthspan [7,8,9].

Recent advances in spatial biology have further enabled precise localization of senescence-associated signals in tissues [10,11]. Multiplexed Error-Robust Fluorescence In Situ Hybridization (MERFISH) has mapped senescent-like microglia within demyelinating lesions, linking a localized senescence-associated state to impaired remyelination [12]. Targeted in situ transcriptomics profiling using Xenium has also been applied to senescence during tissue repair, identifying a p21-high senescent population in skin wounds and showing that clearing this population accelerates wound closure [13]. However, mapping senescence in vivo remains difficult as SASP programs are often intertwined, and senescence-like inflammatory programs are often overlapping with other stress responses [5]. As a result, approaches that are limited in resolution or that rely on pre-selected marker panels (protein or RNA) can fail to unravel the full diversity of senescent states across complex lineages and microenvironments.

To address these gaps, we developed a fluorescence-guided spatial transcriptomic framework, branching out from our standard Seq-Scope procedure with DAPI-based single-cell segmentation to apply to a well-established p21-Cre dual-reporter mouse model [7,8,13,14,15]. Seq-Scope is a micrometer-resolution spatial transcriptome profiling technology that unbiasedly captures whole tissue transcriptome with high-fidelity mapping of transcriptional diversity [14,15]. This technological combination enables us to (i) precisely localize p21-reporter-positive cells in situ, (ii) resolve their cellular identities and transcriptional states without restricting analysis to a limited marker panel, and (iii) systematically characterize the local cellular neighborhood surrounding these p21-associated cells.

Using this approach in aged liver, we reveal that p21^+^ cells are rare yet broadly distributed, span multiple lineages, and include morphologically similar niches that nonetheless exhibit distinct, cell identity–dependent SASP programs, while also identifying an ISG-enriched stress program that shows limited overlap with p21 positivity, suggesting additional heterogeneity in aging-associated responses.

## 2. Materials and Methods

### 2.1. p21-Cre Dual-Reporter Mice Model

p21-Cre dual-reporter mice on C57BL/6 background were generated as previously described [7,8,13]. In brief, a p21 promoter fragment driving a Cre recombinase and GFP reporter were synthesized by GenScript and cloned into the pBT378 vector for site-specific recombination at the mouse H11 genomic locus. Positive embryonic clones were implanted into C57BL/6 females to generate p21-Cre-GFP mice. p21-Cre-GFP mice were then crossed with floxed knock-in *tdTomato* mice (strain no. 007914, The Jackson Laboratory, Bar Harbor, ME, USA) bearing a loxP-stop-loxP *tdTomato* cassette under the control of the CAG promoter. The offspring, p21-Cre-GFP/*tdTomato* mice (p21-Cre dual-reporter mice), were validated and maintained according to Institutional Animal Care and Use Committee (IACUC) protocol. Three 30-month-old littermates, including two males and one female, were assigned for the following experiments.

### 2.2. Seq-Scope Array Preparation (1st-Seq)

Seq-Scope array was prepared as described previously [14,15,16]. Seq-Scope is a solid phase transcriptome capturing system built upon an Illumina sequencing platform (HiSeq2500 in this study, San Diego, CA, USA) with synthetic barcoded single-stranded oligonucleotide library (HDMI32-DraI, Eurofins, Louisville, KY, USA). 100 pM HDMI32-DraI oligonucleotides were seeded onto the flow cell and amplified through synthesis cycles to form barcoded clusters on the surface. Custom sequencing primer (Read1-DraI, Eurofins) was used at the sequence-by-synthesis stage to read the barcode sequence. Sequencer was manually set to stop after 37 bp single-end reading. Following the run, the flow cell and the output data were retrieved. The output FASTQ file will provide the sequence and the XY coordinates of the barcodes.

The retrieved flow cell requires preprocessing to become Seq-Scope array. The flow cell was washed with nuclease-free water three times initially, followed by overnight incubation of DraI (R0129, NEB, Ipswich, MA, USA) and CIAP (M0525, NEB) enzyme mixture at 37 °C. The flow cell was treated with exonuclease I (M2903, NEB) cocktail at 37 °C for 45 min the next day to remove remaining flow cell-attached primer. The flow cell was washed three times with nuclease-free water, three times with 0.1 N NaOH (5 min each), and three times with 0.1 M Tris (pH 7.5). The cover was then disassembled with a tungsten carbide tip scriber (IMT-8806, IMT, Inami, Wakayama, Japan), exposing the array surface for tissue attachment.

### 2.3. Tissue Preparation, Sectioning, and Processing

Livers dissected from the reporter mice were temporarily fixed in 4% formaldehyde (15170, Electron Microscopy Sciences, Hatfield, PA, USA) and transferred to 30% sucrose for overnight incubation. Tissues were then snap-frozen in liquid nitrogen pre-chilled 2-Methylbutane (MX0760, Sigma-Aldrich, St. Louis, MO, USA) for long-term preservation. Before cryosectioning, frozen tissues were embedded into OCT compound (23-730-571, Fisher Scientific, Waltham, MA, USA). Sectioning was performed on a cryostat (CM3050S, Leica, Nussloch, Germany, −15 °C) at a 5° cutting angle to obtain 10 µm sections. Sections were placed on the array surface and re-warmed at room temperature for complete attachment. Attached sections were again fixed in 4% PFA at room temperature for 10 min. Fixing solution was washed away with nuclease-free water, followed by 10 min room temperature 0.1 µg/mL DAPI solution (62248, Thermo Fisher Scientific, Waltham, MA, USA) incubation. Stained tissue sections were mounted in 85% glycerol (BP229-1, Fisher Scientific) with coverslips for fluorescence imaging using Keyence digital darkroom system. Afterward, array was retrieved, cleaned, and subjected to hematoxylin and eosin (H&E) staining that was described previously [14,15]. H&E images were also captured by the Keyence digital darkroom system.

### 2.4. Seq-Scope RNA Library Generation and Sequencing (2nd-Seq)

Seq-Scope library preparation was described previously in detail [14,15]. In brief, tissue permeabilization was performed at 37 °C using 0.2 U/μL collagenase I (17018-029, Thermofisher, Waltham, MA, USA) for 20 min, and 1 mg/mL pepsin (P7000, Sigma-Aldrich, St. Louis, MO, USA) in 0.1 M HCl for 10 min. The environment of permeabilized tissue sections was calibrated with 1X Maxima RT buffer (EP0751, Thermofisher), followed by overnight incubation with reverse transcription mixture (1X RT buffer (EP0751, Thermofisher), 4% Ficoll PM-400 (F4375-10G, Sigma), 1 mM dNTPs (N0477L, NEB), RNase inhibitor (30281, Lucigen, Middleton, WI, USA), Maxima H-RTase (EP0751, Thermofisher) in a humidified chamber at 42 °C.

On the following day, sections were incubated with Exonuclease I (M2903, NEB) at 37 °C for 45 min to remove unused single-stranded DNA probes. Sections were then digested in tissue lysis cocktail (100 mM Tris pH 8.0, 100 mM NaCl, 2% SDS, 5 mM EDTA, 16 U/mL Proteinase K (P8107S, NEB) at 37 °C for 40 min. Upon the completion of digestion, the array was washed sequentially with nuclease-free water, 0.1 N NaOH (5 min), and 0.1 M Tris pH7.5, three times each. The array was then loaded with library synthesis mixture (1X NEBuffer-2 (NEB), 10 uM TruSeq Read2-conjugated Random Primer (IDT), 1 mM dNTPs (N0477, NEB), Klenow Fragment (M0212, NEB), and nuclease-free water) and incubated in a humidified chamber at 37 °C for 2 h. The array was cleaned with nuclease-free water after the completion of the reaction. The array was then incubated with 0.1 N NaOH for 5 min to elute the barcoded cDNA library. The elution was neutralized by 3 M potassium acetate (pH 5.5) and purified using AMPure XP beads (1.8X bead/sample ratio, A63881, Beckman Coulter, Brea, CA, USA) according to the manufacturer’s instruction.

Library amplification was carried out in two PCR steps using Kapa HiFi Hotstart Readymix (KK2602, KAPA Biosystems, Wilmington, MA, USA). In PCR1, TruSeq forward and reverse primers were added, and PCR products were purified using AMPure XP beads (1X bead/sample ratio). In PCR2, TruSeq indexing primers were used, followed by AMPure XP-based size selection (0.6X bead/sample ratio). Libraries were assessed on an Agilent 2100 Bioanalyzer (Agilent Technologies, Santa Clara, CA, USA) and re-purified when necessary before sequencing with paired-end 100-cycle reads.

### 2.5. Sequencing Data Processing

Data were processed as described previously using NovaScope pipeline [14,15]. A 1st-Seq data table was first generated from the 1st-Seq FASTQ files, containing filtered barcode sequences and their corresponding spatial coordinates. The 2nd-Seq FASTQ reads were then processed against this table by (i) mapping reads to the spatial barcode map using the spatial barcode sequences, (ii) filtering barcoded reads based on validated barcode sequences, and (iii) assigning spatial coordinates to the retained reads. Reads with assigned spatial information were aligned to the reference genome using STAR [17], and the resulting alignments were used to generate a spatial digital gene expression (sDGE) matrix.

### 2.6. Alignment Between Histology and Spatial Dataset

Aligned transcripts in sDGE matrix were plotted spatially based on the coordinates for visualization. sDGE images were then subjected to semi-automatic alignment with H&E image using QGIS (version 3.22.9) georeferencer function.

### 2.7. Single Cell Segmentation

DAPI staining was enhanced to generate a DAPI vector mask. DAPI intensity peaks were used as segment centers, and each point was uniformly expanded by a 6-pixel radius, corresponding to approximately 15 μm, considering that hepatocyte diameter is 20–30 μm, to define local cellular territory for transcript assignment. Because segment expansion proceeded isotropically until neighboring regions met, the resulting boundaries provided an approximate cell segmentation based on nuclear position. This approach does not capture the full range of cell size variability across hepatic cell types and may reduce segmentation accuracy in densely packed regions. Transcripts within each segment were aggregated into a single datapoint, which was treated as a single cell with a given segment ID.

### 2.8. Data Analysis and Visualization

Single-cell spatial DGE matrix was analyzed in the Seurat package [18,19]. Cells with low unique feature counts (nFeature cutoff: 100) were removed to optimize clustering performance. This cutoff was applied uniformly across all segmented cells prior to integration and downstream analyses. Although a uniform threshold can in principle affect small immune cells more strongly than hepatocytes, we did not observe an apparent depletion of immune cell populations after filtering. Batches were normalized with SCTransform, followed by integration using SCT-normalized values. Principal components were computed using RunPCA, and high-quality components were selected to generate UMAP embeddings. Cells were clustered using FindNeighbors followed by FindClusters. Cell types were annotated based on marker gene expression identified with FindMarkers and FindAllMarkers functions. Data visualization was supported by plotting functions in Seurat and ggplot2. Cell types were projected into spatial plots based on their segment ID using custom python scripts. Spatial gene expression plot was generated with a custom software platform that displays histology and sDGE matrix simultaneously.

### 2.9. Statistics

Cell type annotation was guided with cluster-enriched marker genes identified using the nonparametric Wilcoxon rank-sum test on SCT-normalized data. To identify niche-associated transcriptional changes within annotated cell types, differential expression was then performed separately within each cell type using the same test framework. For each cell type, two contrasts were evaluated: p21^+^ cells versus the remaining cells of the same cell type, and neighboring cells versus the remaining cells of the same cell type. Genes with adjusted *p*-value < 0.05 and positive log_2_FC were considered significant and visualized. Full differential expression results, including log_2_FC and adjusted p-values, are provided in Table S1.

## 3. Results

### 3.1. Overview of the Experiments and Dataset

p21 is a widely used marker associated with senescence, although p21 expression alone does not capture all senescent states [6]. To accurately localize p21-expressing cells in situ, we previously generated a p21-Cre dual-reporter mouse model [7,8,13]. In our prior study, reporter-positive cells showed increased p21 expression together with enlarged cell size, increased SA-β-gal activity, reduced EdU incorporation, and decreased Lamin B1 expression [8]. These p21^+^ cells also accumulated across multiple tissues in 23-month-old mice, whereas minimal labeling was present in 3-month-old mice, and their depletion improved lifespan and physical function [7,8,13].

**Figure 1:**
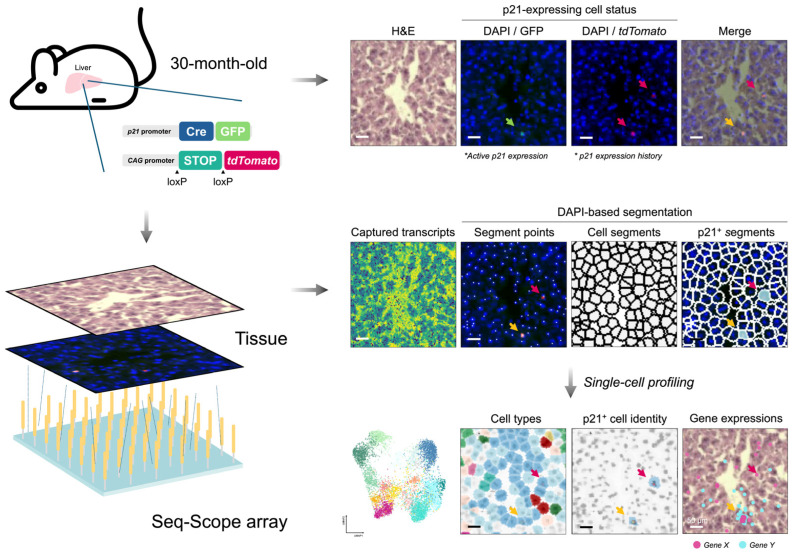
Seq-Scope enables single-cell spatial transcriptome analysis in p21-Cre mouse liver. p21-expressing senescent cells (indicated by colored arrows. Green, GFP; Red, *tdTomato*; Yellow, GFP/*tdTomato*) are visualized in aged p21-Cre-GFP/tdTomato dual-reporter mice (top). Liver sections are profiled by Seq-Scope using a barcoded array and multi-round on-array imaging. DAPI-marked nuclear positions are used as anchor points for cell segmentation and assignment of spatially barcoded reads to individual cells (middle). Cell identities and transcriptional signatures are resolved by dimensionality reduction and clustering of the single-cell spatial transcriptomes.

Here, taking advantage of Seq-Scope’s compatibility with multi-round on-array imaging prior to standard spatial transcriptomic procedures, we were able to collect overall tissue histology, the location of p21-reporting cells, and their cellular transcriptome from the same piece of section. Furthermore, we addressed the challenging cell segmentation in aged tissue, where immune cell infiltration and structural remodeling frequently occur, by anchoring nuclear locations (segment points) using the DAPI layer. Segment points were identified from the DAPI layer and expanded to define approximate local cellular regions for spatial transcriptomic read assignment. Together, we not only identified the major cell types, but also were able to focus on p21^+^ cells to determine their identities and transcriptional programs (Figure 1).

### 3.2. Identification of Liver Zonation and Immune Cell Infiltration in Aged Liver

**Figure 2:**
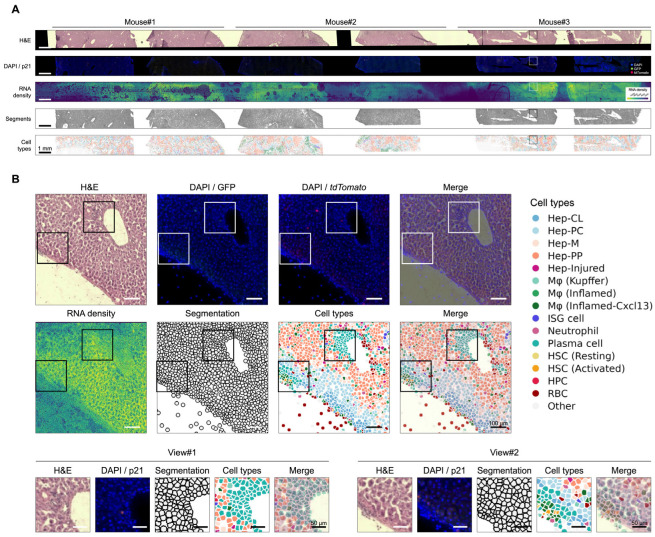
Identification of liver zonation and immune cell infiltration in aged liver. (**A**) Overview of six liver sections from three biological replicates examined by Seq-Scope. From top to bottom, images are displayed as H&E histology, DAPI/GFP/*tdTomato* fluorescence, Seq-Scope RNA density map, DAPI-based segmentation, and spatial cell type projection, according to the clusters identified in Figure 3B. (**B**) Top: spatial maps of boxed region in (**A**). Bottom: higher-magnification views of the corresponding regions in (**B**) top, highlighting plasma cell infiltration (view #1) and macrophage infiltration (view #2).

In this study, six liver sections from three biological replicates of 30-month-old p21-Cre reporter mice were profiled using the Seq-Scope HiSeq2500 platform. The same sections were sequentially subjected to H&E staining (Figure 2A, first row) and DAPI/GFP/*tdTomato* imaging (Figure 2A, second row) on the array surface. Transcripts were then released, spatially barcoded, and collected to construct a sDGE matrix that stores each gene copy along with its spatial coordinates. The sDGE matrix was visualized as an RNA density map (Figure 2A, third row), revealing strong transcript localization to the cytoplasm, consistent with known cellular biology. Overall, 101,532 high-quality segmented cells were identified across all sections.

**Figure 3:**
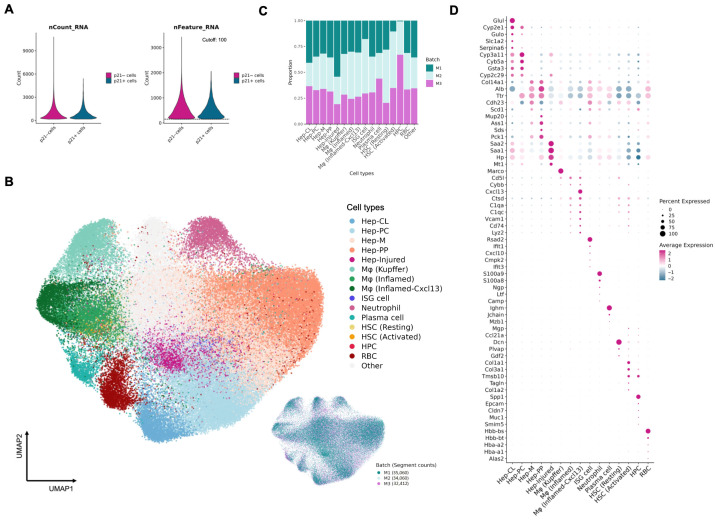
Cell-type landscape of p21-Cre aged mouse liver. (**A**) Distribution of transcript counts (left) and unique RNA features (number of detected genes; right) per segmented cell, shown separately for p21^−^ and p21^+^ cells. A minimum cutoff of 100 unique RNA features was applied prior to integration and downstream analyses. (**B**) UMAP embedding of major cell types identified by multidimensional clustering after integration across mice. Each point (*n* = 101,532) represents one cell and is colored by assigned cell type (center) or by originating mouse (lower right). (**C**) Contribution of each mouse to the indicated cell types. (**D**) Representative expression patterns of cell-type marker genes across clusters.

On average, each segmented cell yielded 475 unique transcripts (Figure 3A). Cells from each mouse were treated as independent groups for integration, followed by clustering. Output clusters were assigned to cell types based on canonical markers (Figure 3B). Batch effects were minimal and did not dominate any major population structure (Figure 3C). In this dataset, we identified hepatocytes (Hep), macrophages (M_Φ_), neutrophils, plasma cells, hepatic stellate cells (HSCs), and hepatic progenitor cells (HPCs), with several populations exhibiting subtype structure. Hepatocytes were further subclustered into zone-specific populations ranging from Periportal (PP), Midzone (M), and Pericentral (PC), to Centrilobular (CL). A distinct hepatocyte subset with high *Saa1* and *Saa2* expression was also identified (Figure 3D; Hep-Injured), suggesting an injury-associated, acute-phase, stress-responsive state [20,21,22]. Macrophages could first be separated into resting and activated states, with the activated population expressing *Vcam1*, *Cd74*, and *H2-Ab1*. Surprisingly, building on this separation, we further identified an activated macrophage subset with high *Cxcl13* expression, indicating layered heterogeneity beyond the resting–activated split in aged liver. In addition, a distinct cell group enriched for type I interferon-stimulated gene (ISG) program was detected, with expression of interferon-associated genes such as *Rsad2*, *Ifit1*, *Cmpk2*, and *Ifit3* (Figure 3D) [23,24].

We next mapped the UMAP-defined cell types back to their spatial coordinates in situ (Figure 2A, fourth row). Zoomed-in views highlighted the organization of major anatomical landmarks, including the portal and central veins, and showed the relative positioning of infiltrating versus resident macrophages, providing a comprehensive spatial view of liver cellular architecture (Figure 2B).

### 3.3. Cellular Identity of p21-Expressing Cells in Aged Liver

**Figure 4:**
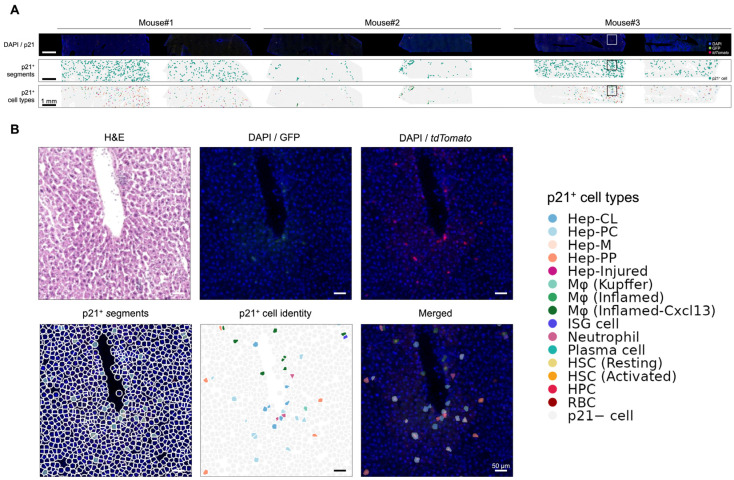
p21-Cre fluorescence guides cell-type assignment of senescent cells. (**A**) Overview of Seq-Scope-profiled liver sections with a focus on p21^+^ cells. From top to bottom, images are displayed as DAPI/GFP/*tdTomato* fluorescence, p21^+^ segments, and spatial projection of p21^+^ cell types based on the clusters defined in Figure 5B. (**B**) Higher-magnification spatial maps of the boxed regions in (**A**).

**Figure 5:**
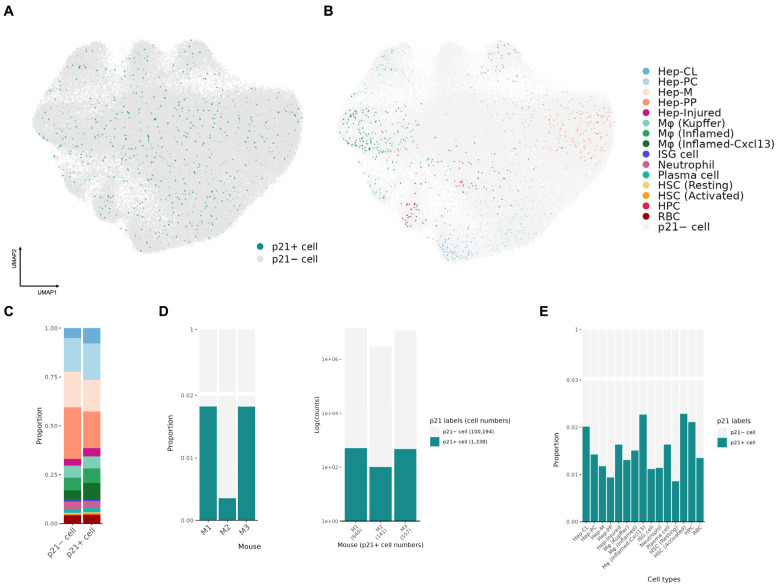
Senescence emerges in parallel across multiple liver lineages in aged liver. (**A**,**B**) UMAP embedding highlighting p21^+^ cells (**A**) or coloring p21^+^ cells by the cell-type clusters defined in Figure 3B (**B**). (**C**) Distribution of cell types within the p21^−^ and p21^+^ cell groups. (**D**) Frequency of p21 positivity across mice, shown as a ratio (left) and as log(counts) (right). (**E**) Frequency of p21 positivity across cell types, shown as a ratio.

To further reveal the identity of reporter-positive p21-associated cells within the whole aged liver population, we isolated segments with overlapping GFP or tdTomato fluorescence (Figure 4A, first and second rows). As a result, 1338 segmented cells were identified as reporter-positive p21-associated cells, including cells positive for both GFP and tdTomato as well as cells positive only for tdTomato, suggesting that the analyzed population included cells with ongoing p21 reporter activity and cells with prior p21 activation history. These cells were largely distributed across the tissue area, with a few exceptions forming local aggregates (Figure 4B). Through cell type identification, we found that p21-reporter-positive cells were distributed across all major cell types (Figure 4B and Figure 5A,B), without a single population dominating the p21^+^ cell group (Figure 5C). The cell type composition of p21^+^ cells was largely similar to the whole population (p21^−^ cells). This diverse-lineage pattern resonates with our previous single-cell RNA-seq results in visceral fat and further supports the conclusion across organs and platforms [7].

By quantifying p21^+^ cells across three biological replicates, we observed discrepancies in senescence occurrence among samples (Figure 5D), but p21^+^ cells remained rare in every sample and the overall multi-lineage distribution was qualitatively consistent across mice. Because the number of p21^+^ cells within individual lineages was limited in each replicate, the present study should be viewed primarily as a proof-of-concept spatial mapping study rather than a formal replicate-by-replicate analysis of all lineage-specific programs. In general, over 50% of p21^+^ cells were hepatocytes, as expected given their dominance in the liver cellular population (Figure 5C,E). Among non-parenchymal cells, macrophages showed the highest tendency to exhibit a p21^+^ state (Figure 5E), consistent with our visceral fat dataset [7]. Notably, we further observed differences in senescence development across macrophage states, with *Cxcl13*-expressing inflamed macrophages showing the highest p21^+^ ratio, nearly twice that of resident macrophages (Kupffer) (Figure 5E). Activated HSCs also exhibited a higher tendency to express p21 compared with resting HSCs, although the sample size was small and additional validation may be needed (Figure 5E).

Overall, these results suggest that p21-associated cell states arise across multiple cell types and that cellular state may influence their distribution in non-parenchymal populations.

### 3.4. Distinct SASP Programs Reveal Heterogeneity Among p21-Expressing Senescent Cells

Cellular senescence is widely defined not only by stable cell-cycle arrest, but also by acquisition of a senescence-associated secretory phenotype, a characteristic program that includes inflammatory signaling, extracellular matrix remodeling, and other paracrine signals [4]. Given this complexity, even a single cell type can adopt distinct SASP programs across different microenvironments.

**Figure 6:**
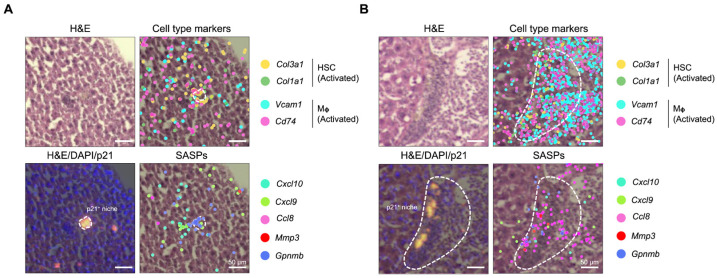
Distinct SASP programs reveal heterogeneity among *p21*-expressing senescent cells. (**A**,**B**) Two p21^+^ senescence niches are shown. Panels display histology (top left), an H&E/DAPI/GFP/tdTomato overlay (bottom left) with the p21^+^ niche outlined by a white dashed boundary, cell-type marker expression (top right), and selected SASP genes (bottom right). Genes are plotted as color dots.

Here, by leveraging the high-resolution feature of Seq-Scope, we scanned the transcriptome of each p21^+^ cell individually in the tissue. We identified two morphologically similar cell aggregates within the p21^+^ niche that nevertheless expressed two distinct SASP programs (Figure 6A,B). Based on histology, both niches appeared as non-parenchymal cell aggregates. Transcriptome data further pointed to their identities as activated HSCs and macrophages, supported by mixed local expression of *Col1a1*, *Col3a1*, *Vcam1*, and *Cd74* (Figure 6A,B, top row). Their SASP signatures, however, differed. While one niche predominantly expressed *Gpnmb* (seno-antigen) [25,26], surrounded by inflammatory SASPs such as *Cxcl9* and *Cxcl10* (Figure 6A, bottom row), the other niche more prominently expressed *Ccl8* and *Mmp3*, reflecting SASP programs associated with inflammation and tissue remodeling, with less pronounced expression of *Gpnmb* (Figure 6B, bottom row). By visualizing the spatial expression of selected SASP genes, these two otherwise similar microenvironments were shown to be transcriptionally distinct, indicating heterogeneity of senescence even within the same p21^+^ niche.

### 3.5. Cell Types Exhibit Distinct Transcriptome Profiles Within the Senescent Niche

**Figure 7:**
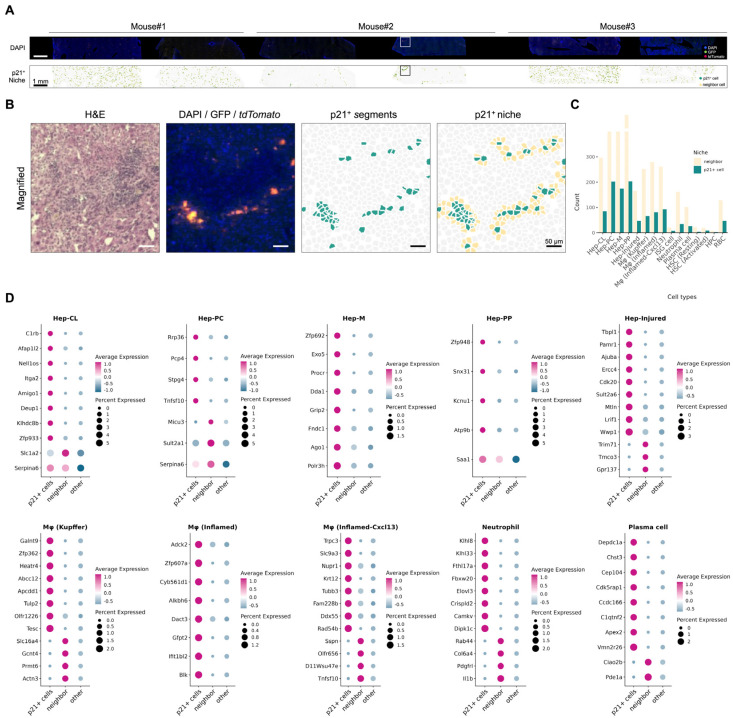
Cell types exhibit distinct transcriptome profiles within the senescent niche. (**A**) Overview of Seq-Scope–profiled liver sections with a focus on p21^+^ niches. From top to bottom, images are displayed as DAPI/GFP/*tdTomato* fluorescence and cells residing within p21^+^ niches. (**B**) Higher-magnification spatial maps of the boxed regions in (**A**). p21^+^ cells (green), neighboring cells (yellow), and other cells (gray) are shown in the right two panels. (**C**) Quantification of the prevalence of p21^+^ and neighboring cells across cell types. (**D**) Expression patterns of genes selectively upregulated in p21^+^ cells or in neighboring cells across cell types (adjusted *p*-values < 0.05).

To further investigate the senescence microenvironment, we collected neighboring cells defined as segmented cells whose boundaries directly contacted a p21^+^ cell segment (Figure 7A, bottom row; Figure 7B). In total, 4902 neighboring cells were isolated. The cell-type composition of these neighboring cells was similar to that of the p21^+^ population, with no single cell type dominating (Figure 7C).

We next performed differential expression (DE) analyses comparing p21^+^ cells, neighboring cells, and other cells within each cell type to identify spatially patterned transcriptional changes associated with the senescent niche (Figure 7D). This analysis revealed position- and lineage-specific enrichment of genes that match to canonical senescence-related programs, including inflammatory sensing, proteostasis remodeling, and metabolic stress adaptation (Table S1).

In parenchymal populations, Hep-CL p21^+^ cells showed significant enrichment of *Itga2* [27] and *C1rb* [28], suggesting altered ECM interaction and complement-associated inflammatory programs. Hep-M p21^+^ cells were marked by elevated *Lcn2*, a well-established hepatocyte injury-response gene [29], together with *Procr*, which may reflect a stress-associated or transitional state [30]. Hep-PC p21^+^ cells upregulated *Tnfsf10*, suggesting the presence of inflammatory and death-receptor signaling [31,32].

Among immune-lineage populations, p21^+^ inflamed *Cxcl13*^+^ macrophages showed significant enrichment of *Gpnmb*, *Kcnn4* [33], *Nupr1* [34], and *Scimp* [35], suggesting an injury-responsive macrophage state involving stress adaptation and inflammatory regulation. In contrast, p21^+^ cell-neighboring p21^−^ *Cxcl13*^+^ macrophages showed significant upregulation of *Tnfsf10*, suggesting a distinct inflammatory or injury-associated program.

In neutrophils, p21^+^ cells were enriched for *Crispld2* [36], *Dpep2* [37], *Gzma* [38], and *Lum* [39], suggesting altered inflammatory and stress-associated features. By contrast, neighboring neutrophils showed significant upregulation of *Il1b*, suggesting more overt pro-inflammatory program [40]. In plasma cells, p21^+^ cells showed increased *Clic1* [41] together with *Mgst2* [42], which may reflect oxidative or stress-related changes.

Together, the DE result indicated that p21^+^ cells and their immediate neighbors engage distinct, cell-type-specific programs that align with senescence-associated stress, inflammatory, and remodeling axes rather than a single shared phenotype restricted to only the senescent cell itself (Figure 7D).

### 3.6. Interferon-Stimulated Gene Activation in Aging Shows Limited Overlap with p21 Positivity

Interferon-stimulated genes are a transcriptional program induced downstream of type I interferon signaling that can be activated not only by infection, but also by DNA damaging stress [23,24]. It has been reported that ISG activity is a recurrent component of senescence biology, with roles in mediating DNA damage response and shaping inflammatory signaling [24,43].

**Figure 8:**
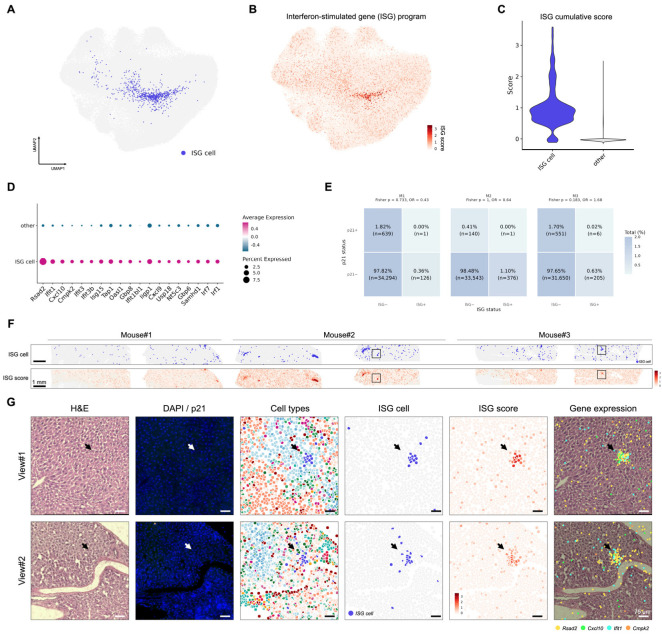
Interferon-stimulated gene activation in aging shows limited overlap with p21 positivity. (**A**,**B**) UMAP embedding colored by the ISG cluster (blue) (**A**) or by the cumulative ISG score (red) (**B**). (**C**) Quantification of the cumulative ISG gene score. (**D**) Significantly upregulated ISGs (adjusted *p*-values < 0.05, Log_2_FC > 1.5). (**E**) Heatmap quantifying overlap between p21 status (p21^+^ or p21^−^) and the ISG status (ISG^+^ or ISG^−^). Tiles show the percentage of total cells in each category, with raw counts indicated. Color scaling was capped to improve visualization of rare populations. Fisher’s exact test was performed on each replicate-specific 2 × 2 contingency table. (**F**) Overview of liver sections colored by ISG cluster (top row) or by cumulative ISG gene score (bottom row). (**G**) Higher-magnification spatial maps of the boxed regions in (**F**). Arrows indicate ISG cells. Expression of the indicated ISG genes is shown as color dots in gene-expression maps.

In our aged liver dataset, we were able to identify this ISG-expressing cluster (Figure 8A) as well, with a distinct transcriptional signature. We summarized this program using a cumulative ISG score and assessed its relative expression across cells (Figure 8B,C). The individual ISGs contributing to the score are shown in Figure 8D, with *Rsad2* exhibiting the most pronounced change. In addition to canonical ISGs, inflammatory chemokines such as *Cxcl10* and *Cxcl9* were also upregulated in this cluster, suggesting a locally activated inflammatory niche.

Spatial mapping further revealed aggregated foci of ISG-expressing cells (Figure 8E–G) in the aged liver. Based on histology, these cells are likely hepatocytes, despite reduced expression of canonical hepatic markers (Figure 3D and Figure 8G). Notably, p21^+^ cells showed relatively low overlap with the ISG-expressing population by fluorescence and cell-type matching (Figure 5E and Figure 8E–G). Consistent with this observation, Fisher’s exact test performed in each biological replicate did not show significant enrichment of p21 positivity within the ISG-enriched population, and p21^+^/ISG^+^ double-positive cells were rare (Figure 8E), supporting the interpretation that these two populations have limited overlap.

## 4. Discussion

Senescence has long been a central focus in gerontology, given its complex cell-intrinsic and intercellular features and its broad contributions to aging-associated degeneration and disease [1]. Yet, despite advances in multi-omic profiling, defining senescent states in vivo remains challenging as senescent cells are both rare and highly heterogeneous [5,10]. By combining micrometer-resolution spatial transcriptomics with DAPI-based single-cell segmentation in a p21-Cre dual-reporter mouse model, we establish a framework to localize p21-lineage cells in situ and resolve their transcriptomes within intact aged tissue. Together, the system enables a comprehensive analysis that compares senescent cells across lineages and within senescent microenvironments.

Our findings are broadly consistent with prior aging atlas studies, including Tabula Muris Senis and a recent human liver single-cell aging atlas, which similarly suggest that aging-associated and senescence-related cell states are distributed across multiple parenchymal and non-parenchymal liver populations and are accompanied by inflammatory and stress-associated transcriptional programs [10,44,45]. The transcriptional changes we detect in cells immediately adjacent to p21^+^ cells support prior observations that senescence is not purely an intercellular program, but can propagate stress, inflammatory, and remodeling signals into the surrounding niche [45]. These comparisons are intended as qualitative context rather than formal external validation, because we did not perform cross-dataset integration or reanalysis.

Building on these shared themes, our reporter-guided spatial framework uncovers additional layers of organization that are difficult to resolve with dissociation-based or marker-limited approaches. We identify morphologically similar non-parenchymal aggregates that nonetheless exhibit distinct SASP programs, indicating that senescent niches can be transcriptionally heterogeneous even when their histological appearance is comparable. By explicitly contrasting p21^+^ cells with their immediate neighbors within each lineage, we distinguish putative “providers” of senescence-associated signals (the senescent cells) from “receivers” (their neighboring cells), revealing that these niche-associated responses are highly cell type–dependent rather than a single shared signature. Finally, we identify an ISG-enriched cellular program with limited overlap with p21 positivity, suggesting that these two programs can arise separately in aged liver, although their mechanistic relationship remains unresolved.

Despite the informative discoveries of this study, several limitations should be acknowledged. First, the regulation of senescence extends beyond transcription, and prior work has reported cases in which transcriptomic changes do not align with proteomic readouts [11,46,47,48]. It therefore remains unclear whether the mRNA differences we observe can be translated into corresponding changes in protein abundance. Second, because p21 reporter positivity was used here to localize and profile cells in situ, the analyzed population should be interpreted as a senescence-enriched p21-associated population rather than a universally definitive marker of senescence, and future studies incorporating orthogonal validation on the same tissue sections will be important. In addition, because reporter-positive cells were defined by overlapping GFP or tdTomato fluorescence, the analyzed p21-associated population included both cells with ongoing p21 reporter activity and cells with prior p21 activation history. This likely introduced additional heterogeneity into the downstream transcriptomic analyses of the reporter-positive population. Third, senescence is highly context dependent. Aging trajectories can diverge substantially across individuals due to differences in lifetime exposures and physiological history [1,49,50]. Although we profiled three biological replicates, consisting of two males and one female, and observed qualitatively consistent patterns, the limited cohort size and inclusion of only one female mouse preclude assessment of inter-individual variability or sex-dependent differences; larger, sex-balanced cohorts will be needed to strengthen the generalizability of these findings. Fourth, matched Seq-Scope data from young reporter mice were not included in the present study, so the current dataset should be interpreted as an aged-liver spatial mapping framework rather than a cross-age comparison of baseline and aging-associated states. Finally, aging biology differs between rodents and humans, and findings from a mouse model may not fully translate to human liver [5,51,52]. Future studies in human tissue will be essential to validate these observations and define their relevance in human aging. Still, as an exploratory study, our framework illustrates the complexity and heterogeneity of senescence across cell types and their spatial niches.

## 5. Conclusions

We developed a fluorescence-guided spatial transcriptomic framework that integrates Seq-Scope with DAPI-based single-cell segmentation and a p21-Cre dual-reporter system to localize p21-lineage cells in situ and profile their transcriptomes and local neighborhoods at micrometer resolution. Applying this approach to aged liver, we show that p21^+^ cells are rare yet distributed across multiple lineages, exhibit cell identity-dependent SASP heterogeneity, and induce morphologically similar yet transcriptionally distinct microenvironments. In parallel, we identify an ISG-enriched cellular state with limited overlap with p21 positivity, further illustrating the heterogeneity of aging-associated cellular programs.

## Data Availability

All source data, source codes for custom analyses in the paper and processed datasets were deposited to the Deep Blue repository (https://doi.org/10.7302/vj8s-t515).

## Supplementary Materials

The following supporting information can be downloaded at: https://www.mdpi.com/article/10.3390/biom16040579/s1, Table S1: Cell type-specific differential expression results for p21^+^ cells and neighboring cells versus the remaining cells of the same cell type in aged liver Seq-Scope data, including log2 fold changes and adjusted p-values.

## Abbreviations

The following abbreviations are used in this manuscript:

SASP	Senescence-associated secretory phenotype
ISG	Interferon-stimulated gene
sDGE	Spatial digital gene expression
Hep	Hepatocytes
M_Φ_	Macrophages
HSC	Hepatic stellate cells
HPC	Hepatic progenitor cells
PP	Periportal
M	Midzone
PC	Pericentral
CL	Centrilobular
DE	Differential expression

## References

[1] Gorgoulis V., Adams P.D., Alimonti A., Bennett D.C., Bischof O., Bishop C., Campisi J., Collado M., Evangelou K., Ferbeyre G. (2019). Cellular Senescence: Defining a Path Forward. Cell.

[2] Gasek N.S., Kuchel G.A., Kirkland J.L., Xu M. (2021). Strategies for Targeting Senescent Cells in Human Disease. Nat. Aging.

[3] Wang B., Han J., Elisseeff J.H., Demaria M. (2024). The senescence-associated secretory phenotype and its physiological and pathological implications. Nat. Rev. Mol. Cell Biol..

[4] Coppé J.-P., Patil C.K., Rodier F., Sun Y., Muñoz D.P., Goldstein J., Nelson P.S., Desprez P.-Y., Campisi J. (2008). Senescence-associated secretory phenotypes reveal cell-nonautonomous functions of oncogenic RAS and the p53 tumor suppressor. PLoS Biol..

[5] Ogrodnik M., Acosta J.C., Adams P.D., di Fagagna F.D., Baker D.J., Bishop C.L., Chandra T., Collado M., Gil J., Gorgoulis V. (2024). Guidelines for Minimal Information on Cellular Senescence Experimentation in vivo. Cell.

[6] Noda A., Ning Y., Venable S.F., Pereira-Smith O.M., Smith J.R. (1994). Cloning of senescent cell-derived inhibitors of DNA synthesis using an expression screen. Exp. Cell Res..

[7] Wang B., Wang L., Gasek N.S., Kuo C.-L., Nie J., Kim T., Yan P., Zhu J., Torrance B.L., Zhou Y. (2024). Intermittent clearance of p21-highly-expressing cells extends lifespan and confers sustained benefits to health and physical function. Cell Metab..

[8] Wang B., Wang L., Gasek N.S., Zhou Y., Kim T., Guo C., Jellison E.R., Haynes L., Yadav S., Tchkonia T. (2021). An inducible p21-Cre mouse model to monitor and manipulate p21-highly-expressing senescent cells in vivo. Nat. Aging.

[9] Wang L., Wang B., Gasek N.S., Zhou Y., Cohn R.L., Martin D.E., Zuo W., Flynn W.F., Guo C., Jellison E.R. (2022). Targeting p21Cip1 highly expressing cells in adipose tissue alleviates insulin resistance in obesity. Cell Metab..

[10] Li S., Garcia P.A.A., Aliferis C., Becich M.J., Calyeca J., Cosgrove B.D., Elisseeff J., Farzad N., Fertig E.J., Glass C. (2025). Advancing biological understanding of cellular senescence with computational multiomics. Nat. Genet..

[11] Gurkar A.U., Gerencser A.A., Mora A.L., Nelson A.C., Zhang A.R., Lagnado A.B., Enninful A., Benz C., Furman D., Beaulieu D. (2023). Spatial mapping of cellular senescence: Emerging challenges and opportunities. Nat. Aging.

[12] Gross P.S., Durán-Laforet V., Ho L.T., Melchor G.S., Zia S., Manavi Z., Barclay W.E., Lee S.H., Shults N., Selva S. (2025). Senescent-like microglia limit remyelination through the senescence associated secretory phenotype. Nat. Commun..

[13] Gasek N.S., Yan P., Zhu J., Purushothaman K.-R., Kim T., Wang L., Wang B., Flynn W.F., Sun M., Guo C. (2025). Clearance of p21 highly expressing senescent cells accelerates cutaneous wound healing. Nat. Aging.

[14] Cho C.-S., Xi J., Si Y., Park S.-R., Hsu J.-E., Kim M., Jun G., Kang H.M., Lee J.H. (2021). Microscopic examination of spatial transcriptome using Seq-Scope. Cell.

[15] Kim Y., Cheng W., Cho C.-S., Hwang Y., Si Y., Park A., Schrank M., Hsu J.-E., Anacleto A., Xi J. (2025). Seq-Scope: Repurposing Illumina sequencing flow cells for high-resolution spatial transcriptomics. Nat. Protoc..

[16] Do T.H., Ma F., Andrade P.R., Teles R., Silva B.J.d.A., Hu C., Espinoza A., Hsu J.-E., Cho C.-S., Kim M. (2022). TREM2 macrophages induced by human lipids drive inflammation in acne lesions. Sci. Immunol..

[17] Dobin A., Davis C.A., Schlesinger F., Drenkow J., Zaleski C., Jha S., Batut P., Chaisson M., Gingeras T.R. (2013). STAR: Ultrafast universal RNA-seq aligner. Bioinformatics.

[18] Hao Y., Hao S., Andersen-Nissen E., Mauck W.M., Zheng S., Butler A., Lee M.J., Wilk A.J., Darby C., Zager M. (2021). Integrated analysis of multimodal single-cell data. Cell.

[19] Stuart T., Butler A., Hoffman P., Hafemeister C., Papalexi E., Mauck W.M., Hao Y., Stoeckius M., Smibert P., Satija R. (2019). Comprehensive Integration of Single-Cell Data. Cell.

[20] Li M., Kim Y.-M., Koh J.H., Park J., Kwon H.M., Park J.-H., Jin J., Park Y., Kim D., Kim W.-U. (2024). Serum amyloid A expression in liver promotes synovial macrophage activation and chronic arthritis via NFAT5. J. Clin. Investig..

[21] Thorn C.F., Lu Z.-Y., Whitehead A.S. (2003). Tissue-specific regulation of the human acute-phase serum amyloid A genes, SAA1 and SAA2, by glucocorticoids in hepatic and epithelial cells. Eur. J. Immunol..

[22] You K., Wang Y., Chen X., Yang Z., Chen Y., Tan S., Tao J., Getachew A., Pan T., Xu Y. (2023). Neutralizing serum amyloid a protects against sinusoidal endothelial cell damage and platelet aggregation during acetaminophen-induced liver injury. Biochem. Biophys. Res. Commun..

[23] Schneider W.M., Chevillotte M.D., Rice C.M. (2014). Interferon-stimulated genes: A complex web of host defenses. Annu. Rev. Immunol..

[24] Yu Q., Katlinskaya Y.V., Carbone C.J., Zhao B., Katlinski K.V., Zheng H., Guha M., Li N., Chen Q., Yang T. (2015). DNA damage-induced type I interferon promotes senescence and inhibits stem cell function. Cell Rep..

[25] Suda M., Shimizu I., Katsuumi G., Yoshida Y., Hayashi Y., Ikegami R., Matsumoto N., Yoshida Y., Mikawa R., Katayama A. (2021). Senolytic vaccination improves normal and pathological age-related phenotypes and increases lifespan in progeroid mice. Nat. Aging.

[26] Hsiao C.L., Katsuumi G., Yoshida Y., Furihata T., Joki Y., Furuuchi R., Liang J.Q., Nakagami H., Minamino T. (2024). Vaccination targeting senescence-associated transmembrane protein alleviated cardiovascular pathology in the mice. Eur. Heart J..

[27] Zeltz C., Gullberg D. (2016). The integrin–collagen connection—A glue for tissue repair?. J. Cell Sci..

[28] Dunkelberger J.R., Song W.-C. (2010). Complement and its role in innate and adaptive immune responses. Cell Res..

[29] Bahmani B., Amiri F., Mohammadi Roushandeh A., Bahadori M., Harati M.D., Habibi Roudkenar M. (2015). The Lcn2-engineered HEK-293 cells show senescence under stressful condition. Iran. J. Basic Med. Sci..

[30] Mosnier L.O., Zlokovic B.V., Griffin J.H. (2007). The cytoprotective protein C pathway. Blood.

[31] Krishnan A., Ozturk N.B., Cutshaw K.A., Guicciardi M.E., Kitagataya T., Olson K.E., Pavelko K.D., Sherman W., Wixom A.Q., Jalan-Sakrikar N. (2024). Tumor necrosis factor-related apoptosis-inducing ligand (TRAIL) deletion in myeloid cells augments cholestatic liver injury. Sci. Rep..

[32] Hirsova P., Weng P., Salim W., Bronk S.F., Griffith T.S., Ibrahim S.H., Gores G.J. (2017). TRAIL deletion prevents liver inflammation but not adipose tissue inflammation during murine diet-induced obesity. Hepatol. Commun..

[33] Kang H., Kerloc’h A., Rotival M., Xu X., Zhang Q., D’sOuza Z., Kim M., Scholz J.C., Ko J.-H., Srivastava P.K. (2014). Kcnn4 is a regulator of macrophage multinucleation in bone homeostasis and inflammatory disease. Cell Rep..

[34] Huang C., Santofimia-Castaño P., Iovanna J. (2021). NUPR1: A Critical Regulator of the Antioxidant System. Cancers.

[35] Luo L., Bokil N.J., Wall A.A., Kapetanovic R., Lansdaal N.M., Marceline F., Burgess B.J., Tong S.J., Guo Z., Alexandrov K. (2017). SCIMP is a transmembrane non-TIR TLR adaptor that promotes proinflammatory cytokine production from macrophages. Nat. Commun..

[36] Zhang S., Pei L., Qu J., Sun L., Jiang W., Li W., Lin Z., Chen D. (2021). CRISPLD2 attenuates pro-inflammatory cytokines production in HMGB1-stimulated monocytes and septic mice. Am. J. Transl. Res..

[37] Zhan Z., Liang H., Zhao Z., Pan L., Li J., Chen Y., Xie Z., Yan Z., Xiang Y., Liu W. (2025). The Trim32-DPEP2 axis is an inflammatory switch in macrophages during intestinal inflammation. Cell Death Differ..

[38] van Daalen K.R., Reijneveld J.F., Bovenschen N. (2020). Modulation of Inflammation by Extracellular Granzyme A. Front. Immunol..

[39] Li Z., Sun C., Chen M., Wang B. (2021). Lumican silencing alleviates tumor necrosis factor-α-induced nucleus pulposus cell inflammation and senescence by inhibiting apoptosis signal regulating kinase 1/p38 signaling pathway via inactivating Fas ligand expression. Bioengineered.

[40] Lau L., Porciuncula A., Yu A., Iwakura Y., David G. (2019). Uncoupling the Senescence-Associated Secretory Phenotype from Cell Cycle Exit via Interleukin-1 Inactivation Unveils Its Protumorigenic Role. Mol. Cell. Biol..

[41] Domingo-Fernández R., Coll R.C., Kearney J., Breit S., O’Neill L.A.J. (2017). The intracellular chloride channel proteins CLIC1 and CLIC4 induce IL-1β transcription and activate the NLRP3 inflammasome. J. Biol. Chem..

[42] Dvash E., Har-Tal M., Barak S., Meir O., Rubinstein M. (2015). Leukotriene C4 is the major trigger of stress-induced oxidative DNA damage. Nat. Commun..

[43] Wang D., Chen K., Wang Z., Wu H., Li Y. (2024). Research progress on interferon and cellular senescence. FASEB J..

[44] Schaum N., Lehallier B., Hahn O., Pálovics R., Hosseinzadeh S., Lee S.E., Sit R., Lee D.P., Losada P.M., Zardeneta M.E. (2020). Ageing hallmarks exhibit organ-specific temporal signatures. Nature.

[45] Karpova A., Li X., Peng C.-W., Gallant K.L., Rapp D.R., Alligood D.M., Houston A.J., Park A., da Costa A.L.N.T., Chou W.-H. (2026). Cellular senescence in human liver under normal aging and cancer. Cell Genom..

[46] Basisty N., Kale A., Jeon O.H., Kuehnemann C., Payne T., Rao C., Holtz A., Shah S., Sharma V., Ferrucci L. (2020). A proteomic atlas of senescence-associated secretomes for aging biomarker development. PLoS Biol..

[47] Vogel C., Marcotte E.M. (2012). Insights into the regulation of protein abundance from proteomic and transcriptomic analyses. Nat. Rev. Genet..

[48] Gygi S.P., Rochon Y., Franza B.R., Aebersold R. (1999). Correlation between protein and mRNA abundance in yeast. Mol. Cell. Biol..

[49] Nielsen L., Marsland A.L., Hamlat E.J., Epel E.S. (2024). New Directions in Geroscience: Integrating Social and Behavioral Drivers of Biological Aging. Psychosom. Med..

[50] Ferrucci L., Wilson D.M., Donega S., Montano M. (2024). Enabling translational geroscience by broadening the scope of geriatric care. Aging Cell.

[51] Demetrius L. (2006). Aging in mouse and human systems: A comparative study. Ann. N. Y. Acad. Sci..

[52] Kõks S., Dogan S., Tuna B.G., González-Navarro H., Potter P., Vandenbroucke R.E. (2016). Mouse models of ageing and their relevance to disease. Mech. Ageing Dev..

